# Preventing Excessive Gestational Weight Gain During Consecutive Pregnancies: Findings from an mHealth Intervention

**DOI:** 10.1007/s10995-026-04247-2

**Published:** 2026-04-04

**Authors:** Anna Roesler, Femke Geusens, Hanne Van Uytsel, Marlien Varnfield, Roland Devlieger, Annick Bogaerts

**Affiliations:** 1 The Australian e-Health Research Centre, CSIRO, Herston, QLD 4006 Australia; 2 Department of Development and Regeneration, Cluster Woman and Child, REALIFE Research Group, KU Leuven, Leuven, Belgium; 3 Department of Women’s and Children’s Health, Uppsala University, Uppsala, Sweden; 4 Department of Obstetrics and Gynaecology, University Hospitals, KU Leuven, Leuven, Belgium; 5 Faculty of Health, University of Plymouth, Plymouth, UK

**Keywords:** EGWG, Pregnancy, Gestational weight gain, mHealth intervention

## Abstract

**Introduction:**

Excessive gestational weight gain (EGWG) poses significant health risks, including increased pregnancy complications, obesity, and metabolic disorders. Addressing EGWG is critical for women with a history of EGWG, as recurrence is common in consecutive pregnancies. This study presents findings from a subanalysis of the intervention arm of the INTER-ACT multi-center randomied controlled trial (RCT), which evaluated the impact of a combined mobile health (mHealth) and face-to-face lifestyle coaching intervention on EGWG recurrence during consecutive pregnancies.

**Methods:**

This subanalysis focuses on the intervention arm of the INTER-ACT RCT, conducted in six hospitals in Flanders, Belgium. The intervention consisted of two phases: the interpregnancy period (six weeks to six months postpartum) and the consecutive pregnancy (before 15 weeks post-conception to birth). Participants (*n* = 172) with a history of EGWG received health coaching and used an app to self-monitor weight, steps, and mental health. A multiple regression model assessed the effects of self-monitoring and coaching attendance on EGWG during the consecutive pregnancy in the intervention arm.

**Results:**

App engagement decreased from 78% during the interpregnancy period to 37% during the consecutive pregnancy. Self-monitoring behaviors (logging weight, steps, and mental health) or coaching attendance did not independently predict EGWG. A prior history of EGWG was the strongest predictor of EGWG recurrence, followed by older maternal age.

**Conclusion:**

While mHealth interventions can promote self-monitoring, sustaining engagement across pregnancies remains challenging. Future strategies should focus on improving long-term adherence to mHealth tools to enhance GWG outcomes and maternal health, especially for women motivated to engage with these technologies. These findings, derived from the intervention arm of the INTER-ACT RCT, suggest that further research is needed to assess the broader applicability of these results.

## Significance

Excessive gestational weight gain (EGWG) is a known risk factor for adverse maternal and child health outcomes, with recurrence common in subsequent pregnancies. Digital health interventions show promise but face engagement challenges. This study adds to the literature by evaluating an mHealth intervention aimed at reducing EGWG recurrence, demonstrating that while some women engaged with the intervention, overall results showed no significant effect. These findings highlight the need for further research to identify which women may benefit, how to maintain engagement, and how app-base strategies can be integrated with broader environmental and behavioral support.

## Introduction

Excessive gestational weight gain (EGWG) is a common occurrence during pregnancy, with over half of women surpassing the recommended gain in weight (Denize et al., 2018). EGWG is associated with adverse outcomes, including maternal weight retention, and pregnancy-and birth related complications in subsequent pregnancies (Chen & Chauhan, 2019). Various interventions, such as coaching, supervised exercise programs, and prescribed dietary programs, have been implemented to help women achieve their GWG recommendations, many of which are initiated during pregnancy (Cantor et al., 2021). These interventions have increasingly included digital components. A scoping review by Raab et al. (2023) examined eHealth and mHealth app-supported lifestyle interventions during pregnancy to manage GWG and prevent gestational diabetes mellitus (GDM). Of the 18 trials identified, 39% reported significant intervention effects on GWG. The reviewed interventions primarily utilised mHealth apps featuring self-monitoring, feedback, goal setting, prompts, and educational content. Notably, only two studies in the Raab et al. (2023) scoping review, including the intervention described herein, looked at the reproductive continuum (interpregnancy/preconception through to pregnancy) and weight intervention.

Overall, there is limited literature on interventions addressing excessive weight gain in pregnancy that target the continuum of preconception, postpartum, and interpregnancy. These periods of time are interrelated and changes in weight during one period can affect the next (Lim et al., 2022), therefore there is a growing consensus that initiating interventions, ideally before conception, is crucial for longer term health outcomes for mother and offspring (Stephenson et al., 2018). Consequently, we developed the INTER-ACT intervention (INTERpregnAncy Coaching for a healthy fuTure) (Bogaerts et al., 2017), which consist of interpregnancy follow-up (phase I) and a consecutive pregnancy intervention (phase II) (Bogaerts et al., 2017).

The aim of this study was to assess the impact of (a) self-monitoring via the INTERACT app and (b) lifestyle coaching session attendance as a part of the INTER-ACT intervention on EGWG.

## Methods

### Study Design

This study conducted secondary analysis on data collected from the INTER-ACT multi-center randomised controlled trial (RCT), with detailed information available in the protocol paper by Bogaerts et al. (2017). The INTER-ACT intervention aimed to support women who had EGWG in their recent pregnancy. The primary objective was to mitigate the risks of pregnancy-induced hypertension, GDM, cesarean section, and large-for-gestational-age babies in subsequent pregnancies through encouraging healthy lifestyle changes tailored to individual needs and overcoming barriers. The intervention comprised two phases: phase (1) the postpartum/interpregnancy phase following a pregnancy with EGWG, spanning from six weeks to six months postpartum, and phase (2) the consecutive pregnancy phase. Note we also refer to the previous pregnancy throughout the paper, and this is the pregnancy immediately before phase 1 of the study.

Participants in the intervention group for phase one underwent four face-to-face, one on one lifestyle coaching sessions, scheduled at approximately 6 weeks, 8 weeks, 12 weeks, and 6 months postpartum (timings of sessions were informed by routine postpartum medical visits, and fitting into the critical early postpartum period) and 3 face to face coaching sessions in the consecutive pregnancy. For phase two (the consecutive pregnancy), women were invited for their first coaching session before 15 weeks, and the second and third coaching sessions were scheduled at 20 and 32 weeks respectively. The coaching sessions covered various topics including dietary habits, nutrition, physical activity, stress management, and social support, utilising tailored informational education and decision-making guidance. Coaches received comprehensive manuals to ensure consistency across sessions.

Additionally, a custom mHealth application (app) was developed for this study and installed on participants’ smartphones during the initial coaching session of phase 1. The app was re-installed for phase 2 with some slight changes for the pregnancy period. The primary objective of this app was to support participants in implementing behavior changes between face-to-face coaching sessions. Within the app, participants could monitor their weight using a Bluetooth-connected weighing scale, track their physical activity using a Bluetooth-connected pedometer, and assess their mental wellbeing by inputting their emotional status and stress levels. Personalized goals could be established and progress monitored within the app. Coaches had access to participants’ app data, enabling them to provide personalized motivational messages and informational tips aligned with participants’ goals. App usage was also discussed during coaching sessions to reinforce its integration into participants’ daily routines. Further details regarding the intervention and app design are available in the study protocol (Bogaerts et al., 2017) and Bogaerts et al. (2020) respectively.

The study received ethical approval from the central medical ethical committee UZ/KU Leuven, and the local ethics committees of the participating sites (i.e. GasthuisZusters ziekenhuizen Antwerpen; Universitair Ziekenhuis Antwerpen; Ziekenhuis Oost-Limburg Genk; Sint-Franciscus Ziekenhuis Heusden-Zolder; Jessa Ziekenhuis Hasselt; Universitair Ziekenhuis Gasthuisberg Leuven). The trial was registered with ClinicalTrials.gov in August 2016 with the Identifier: NCT02989142.

### Participants

Data were collected between May 2017 and July 2019 from participants recruited shortly after childbirth by study midwives across six hospitals in Flanders. Eligible participants were at least 18 years old, had experienced EGWG in that pregnancy according to the IOM guidelines (Institute of Medicine, 2009), did not require complex medical diets, had no history of bariatric surgery or planned surgeries, had no chronic or significant psychiatric disorders, and had not experienced a stillbirth in their previous pregnancy. Trial participants were enrolled in the study, after giving written informed consent for data extraction from the medical records, data collection from the questionnaires and mobile app, and for performing physical measurements until the end of the subsequent pregnancy. For this study, participants from the INTER-ACT intervention were included if they had re-engaged with the INTER-ACT program, either by using the app or by attending a coaching session in the consecutive pregnancy (prior to 15 weeks gestation).

### Measures

#### Weight Variables

Gestational Weight Gain (GWG): Due to variability in the timing of weight measurements between pregnancies, we utilized self-reported pre-pregnancy weight and subtracted it from the mother’s recorded weight at birth to estimate GWG. GWG was calculated both for the previous and consecutive pregnancy.

Excessive Gestational Weight Gain (EGWG): To assess EGWG for the consecutive pregnancy, we calculated the difference between total GWG and the IOM (Institute of Medicine, 2009) recommended maximum weight gain for different pre-pregnancy BMI categories: 18 kg for underweight, 16 kg for normal weight, 11.5 kg for overweight, and 9 kg for obese. Due to a small sample size, we could not stratify by weight category but accounted for pre-pregnancy BMI and weight gain exceeding IOM recommendations. EGWG was calculated both for the previous and consecutive pregnancy.

Postpartum Weight Retention (PPWR): PPWR refers to the amount of weight retained by a participant at 6 months postpartum compared to their weight before their previous pregnancy. PPWR was calculated based on self-reported pre-pregnancy weight and postpartum weight measured by a study nurse.

BMI (and change in BMI): The BMI was based on the mother’s self-reported weight pre pregnancy kg/m2. The Change in BMI was the difference between the previous and consecutive pre-pregnancy BMI.

#### App Use

We measured the frequency of logging weight, steps, and mental health data during two time periods: (1) the interpregnancy period (between 6 weeks and 6 months postpartum), and (2) the consecutive pregnancy (between 0-15 weeks gestation and birth). We also summed the total logs (the combined number of weight, steps and mental health logs) across the two time periods.

#### Coaching Session Attendance

Coaching session attendance was measured as the total number of sessions attended out of six recorded sessions (during the interpregnancy and pregnancy, 3 sessions each). Note, attendance at coaching session of week 8 of interpregnancy was not recorded, as this was an education only session, and only when weight was taken was attendance captured. For the regression analyses, the variable was included as a numeric predictor to reflect the ordered nature of attendance.

#### Control Variables

Mother’s age at the start of the consecutive pregnancy: Extrapolated from the baseline self-reported age.

Parity: The number of times the woman has given birth, prior theconsecuctive pregnancy.

History of Depression and Anxiety: Prior the previous pregnancy, participants were asked whether they had suffered from depressive symptoms (depression) or from intense feelings of anxiety (anxiety) (yes or no). These items captured self-reported history of symptoms. Given the scope of the study and the need to minimize survey burden, longer validated instruments were not administered.

Time between pregnancies: Time between birth and start of the consecutive pregnancy.

### Statistical Analyses

All statistical analyses were conducted using SPSS version 29.0.2.0 (20). Firstly, we explored the composition of our sample using descriptive analyses. Mean, SD, range, frequency and percentages were presented for app use (number of weight, step and mental health logs), coaching session attendance, weight (BMI, EGWG, PPWR, total gestational weight gain, change in BMI between pregnancies) and control variables (age, parity, history of depression and anxiety, time between pregnancies) for the overall sample, and grouped depending on app use. The groups included: (1) participants who logged their weight, steps or mental health in the INTER-ACT app during both the interpregnancy period and the consecutive pregnancy; (2) those who logged only during the interpregnancy period; (3) those who did not log during either period; and (4) those who logged only during the consecutive pregnancy. Due to the small sample size (*n* = 5), the group that logged only during the consecutive pregnancy was excluded from the current descriptive analyses. These groups were created to explore whether differences in logging behavior across the phases of the intervention were associated with other variables. To determine differences in our continuous variables between app usage groups, we conducted a Multivariate Analysis of Variance Analysis (MANOVA) using Pillai’s Trace and listwise deletion, as pairwise deletion is not possible for MANOVA in SPSS. For categorical variables (and non-normally distributed continuous variables), cross-tabulations were performed, and group differences were assessed using Pearson’s Chi-Square test.

To address our research questions, we performed a hierarchical linear regression analysis with three steps (step 1 = control variables, step 2 = app-based logging, step 3 = coaching attendance) and 'EGWG in the consecutive pregnancy' as the dependent variable. Note, all participants enrolled in the intervention arm were included in the analysis, and their level of engagement was treated as a continuous exposure variable. In the regression model, we controlled for variables that exhibited a significant bivariate correlation with the outcome variable of EGWG (included covariates: PPWR, EGWG in the previous pregnancy, mothers age and pre-pregnancy BMI before the previous pregnancy). As our analytic approach was data driven rather than hypothesis driven, and because we did not have strong a priori justification to retain noncorrelated variables (time between pregnancies, parity, history of depression, and history of anxiety), these variables were not included as covariates. Pairwise deletion was used for missing data. We conducted the same hierarchical linear regression analysis, this time using total GWG in the consecutive pregnancy as the dependent variable. This approach accounts for the narrower recommended weight gain margins for overweight and obese women, reducing the risk of inadvertently capturing minor instances of EGWG. The results of this analysis were very similar to those obtained when EGWG was used as the dependent variable. Therefore, we have not presented these results, as they lead to the same conclusion.

## Results

### Descriptive Results

The study included 172 women from the intervention group of the INTER-ACT multi-center RCT, all of whom went on to experience a consecutive pregnancy. Prior to their previous pregnancy, 49.4% of participants had a BMI classified as overweight or obese. This proportion increased to 59.3% at the beginning of the consecutive pregnancy, reflecting an average increase of 1.3 BMI units. The average EGWG for the entire group for the previous pregnancy (prior to phase 1) was 4.65 kg (SD = 4.25) versus the consecutive pregnancy (phase 2) EGWG of 2.60 kg (SD = 3.73). The percentage of participants with EGWG in the consecutive pregnancy was 59.9%, so a reduction from 100% in the previous pregnancy.

During phase 1 (interpregnancy period; ~140 days), most participants (*n* = 134, 78%) interacted with the app by entering at least one health metric (e.g., weight, step count). App use declined markedly in phase 2 (consecutive pregnancy; ~175 days), where only 64 participants (37%) recorded any data.

Engagement with coaching was moderate overall, with 54 participants (31%) attending all six sessions in phase 2. Table 1 summarises the participant characteristics by app use in the interpregnancy and consecutive pregnancy periods. The results of the MANOVA indicated that there were significant differences in our sample depending on app usage (V = 0.93, F[38,218] = 5.00, *p* <.001, η_p_² = 0.47). Specifically, Bonferroni posthoc multiple comparison tests demonstrated that women who logged across both phases differed on app-specific engagement variables (greater logging and coaching attendance) and on a contextual characteristic (a shorter interpregnancy interval).

**Table 1:** Participant characteristics and app usage during interpregnancy and consecutive pregnancy periods

Variable	Full analytical sample (*n* = 172)	Logged inter- and logged consecutive- pregnancy (*n* = 59)	Logged inter- and not logged consecutive- pregnancy (*n* = 75)	Not logged inter- or consecutive- pregnancy (*n* = 33)	Test of between-subjects effects
	Mean (SD, range)	Mean (SD, range)	Mean (SD, range)	Mean (SD, range)	*p* ^a^
Weight					
*Interpregnancy*					
BMI before previous pregnancy (kg/m^2^)	25.24 (4.01, 18.67–41.02)	24.98 (4.01, 18.87–35.38)	25.52 (4.11, 18.67–40.83)	24.95 (4.00, 20.70–41.02)	0.97
Previous pregnancy total gestational weight gain (kg)	18.11 (3.93, 10.00–31.80)	18.45 (4.07, 11.60–31.80)	18.02 (3.78, 10.00–30.00)	18.17 (4.10, 10.00–30.00)	0.90
Previous pregnancy EGWG (kg)	4.65 (4.25, 0.00–20.30)	5.19 (4.76, 0.10–20.3)	4.63 (4.21, 0.00–18.50)	3.95 (3.54, 0.50–14.00)	0.33
Previous pregnancy PPWR (kg)	4.45 (5.30; − 10.90 to 20.80)	4.03 (5.03, − 7.20 to 18.30)	5.31 (5.61, − 4.00 to 20.80)	3.89 (5.65, − 10.90 to 18.50)	0.37
*Consecutive pregnancy*					
BMI prior consecutive pregnancy (kg/m^2^)	26.55 (4.53, 17.85–44.29)	26.41 (4.77, 19.29–40.65)	26.99 (4.75, 17.85–44.29)	25.64 (3.72, 18.65–38.28)	0.69
Average change in BMI between previous and consecutive pregnancy (kg/m^2^)	1.31 (1.74, − 2.74 to 9.28)	1.43 (2.03, − 2.57, 9.28)	1.47 (1.58, − 1.90 to 5.58)	0.69 (1.42, − 2.74 to 3.50)	0.12
Total gestational weight gain consecutive pregnancy (kg)	14.01 (5.23, − 7.40 to 29.60)	13.65 (4.66, 2.40–25.00)	14.11 (5.98, − 7.40 to 29.00)	14.67 (4.62, 6.20–29.60)	0.88
EGWG during consecutive pregnancy (kg)	2.60 (3.73, 00–20.60)	2.11 (3.25, 0.00–16.00)	3.02 (3.89, 0.00–20.00)	2.80 (4.31, 0.00–20.60)	0.44
*App use*					
*Interpregnancy*					
Body weight (app logs)	26.28 (32.18, 0–145)	46.05 (35.97, 1–145)	24.04 (26.60, 0–123)	0	< 0.001
Steps (app logs)	43.56 (44.23, 0–146)	70.66 (40.40, 0–143)	44.32 (41.00, 0–146)	0	< 0.001
Mental health (app logs)	14.45 (19.97, 0–98)	25.56 (24.43, 0–98)	13.03 (15.68, 0–84)	0	< 0.001
*Consecutive pregnancy*					
Body weight (app logs)	6.06 (17.81, 0–137)	15.54 (26.17, 0–137)	0	0	< 0.001
Steps (app logs)	8.71 (27.02, 0–221)	22.97 (40.97, 0–221)	0	0	< 0.001
Mental health (app logs)	2.83 (7.53, 0–49)	7.64 (11.12, 0–49)	0	0	< 0.001
Coaching attendance	4.09 (1.68, 1–6)	5.51 (0.73, 3–6)	3.21 (1.50, 1–6)	3.39 (1.64, 1–6)	<.001^b^
Control variables					
Age (years)	30.77 (3.33, 21–41)	30.69 (3.49, 21–41)	30.72 (3.48, 23–39)	31.12 (2.84, 24–36)	0.98
Parity					
1 n (%) 2 n (%) 3 n (%) 4 n (%)	145 (84.3) 23 (13.4) 3 (1.7) 1 (0.6)	51 (86.4) 7 (11.9) – 1 (1.7)	63 (84) 11 (14.7) 1 (1.3) –	26 (78.8) 5 (15.2) 2 (6.1) –	.35^b^
History of depression n (%)	14 (8.5)	4 (7.3)	7 (9.5)	3 (10)	.88^b^
History of anxiety n (%)	16 (9.7)	5 (8.9)	4 (5.4)	6 (20)	.07^b^
Time between pregnancies (months)	18.74 (7.93, 4.79–41.08)	16.53 (7.42, 5.18–41.08)	19.96 (7.53, 4.79–37.15)	19.36 (9.01, 5.93–38.89)	< 0.01

*BMI* body mass index, *PPWR* postpartum weight retention, *EGWG* excessive gestational weight gain

^a^MANOVA uses listwise deletion, resulting in a changed sample size (*n* = 52 interpregnancy + consecutive pregnancy logging, *n* = 53 interpregnancy but not consecutive pregnancy logging, *n* = 24 no logging)

^b^Non-continuous (and non-normally distributed) variables were excluded from the MANOVA. Instead, cross-tabulations were performed using Pearson’s Chi-Square test, and the reported p-values correspond to this analysis

Table 2 presents detailed results of the multiple regression model for predicting EGWG. The covariates (PPWR, EGWG in the previous pregnancy, mother’s age and BMI) accounted for 19.3% of the variance in EGWG. Neither adding app use variables (weight, steps, or mental health) nor the coaching sessions to the model increased the explained variance. Based on the final model, only mother’s age and EGWG in the previous pregnancy were significantly associated with EGWG in the consecutive pregnancy, whereby older mothers and mothers with greater EGWG in the previous pregnancy had greater EGWG in the consecutive pregnancy. In contrast to our expectations, neither app use, nor coaching session attendance were associated with EGWG in the consecutive pregnancy.

**Table 2:** Results of the multiple regression analysis predicting excessive gestational weight gain in the consecutive pregnancy

Predictor	Model I – control variables only				Model II – app use				Model III – coaching session attendance			
	B(SE)	β	t	95%CI	B(SE)	β	t	95%CI	B(SE)	β	t	95%CI
Intercept	− 6.18(3.12)*		− 1.98	[− 12.36, − 0.01]	− 6.37(3.23)		− 1.97	[− 12.76, 0.02]	− 5.55(3.27)		− 1.70	[− 12.02, 0.91]
Pre-pregnancy BMI at study start	0.08(0.08)	0.09	1.02	[− 0.08, 0.24]	0.09(0.08)	0.10	1.09	[− 0.07, 0.25]	0.07(0.08)	0.08	0.86	[− 0.09, 0.23]
EGWG in previous pregnancy	0.27(0.09)**	0.31	3.13	[0.10, 0.44]	0.27(0.09)**	0.30	3.02	[0.09, 0.44]	0.28(0.09)**	0.32	3.18	[0.11, 0.46]
Post partum weight retention	0.06(0.06)	0.09	0.94	[− 0.07, 0.19]	0.06(0.07)	0.09	0.94	[− 0.07, 0.19]	0.04(0.07)	0.06	0.65	[− 0.09, 0.18]
Mothers age – start of consecutive pregnancy	0.17(0.09)	0.15	1.95	[− 0.003, 0.34]	0.18(0.09)	0.16	1.94	[− 0.003, 0.35]	0.20(0.09)*	0.18	2.16	[0.02, 0.38]
Weight logs interpregnancy					0.01(0.01)	0.07	0.68	[− 0.02, 0.03]	0.01(0.01)	0.08	0.80	[− 0.01, 0.03]
Step logs interpregnancy					− 0.01(0.01)	− 0.06	− 0.56	[− 0.02, 0.01]	− 0.003(0.01)	− 0.04	− 0.36	[− 0.02,0.02]
Mental Health logs interpregnancy					0.000(0.02)	− 0.003	− 0.02	[− 0.04, 0.04]	− 0.001(0.02)	− 0.004	− 0.04	[− 0.04,0.04]
Weight logs in consecutive pregnancy					0.001(0.03)	0.003	0.03	[− 0.05, 0.05]	0.004(0.03)	0.02	0.16	[− 0.05, 0.05]
Step logs in consecutive pregnancy					− 0.004(0.02)	− 0.03	− 0.22	[− 0.04, 0.03]	− 0.004(0.02)	− 0.03	− 0.22	[− 0.04, 0.03]
Mental Health logs in consecutive pregnancy					− 0.04(0.07)	− 0.09	− 0.67	[− 0.17, 0.09]	− 0.03(0.07)	− 0.07	− 0.53	[− 0.16, 0.10]
Coaching session attendance									− 0.28(0.20)	− 0.13	− 1.43	[− 0.67, 0.11]
Model fit	*R* =.44, R²_adjusted_ = 0.17, R²_unadjusted_ = 0.19, F(4,136) = 8.11, *p* <.001				*R* =.46, R²_adjusted_ = 0.15, ∆R² = 0.02, ∆F(6,130) = 0.44, *p* =.85				*R* =.47, R²_adjusted_ = 0.15, ∆R² = 0.01, ∆F(1,129) = 2.03, *p* =.16			

* *p* <.05, ** *p* <.01, *** *p* <.001, BMI = body mass index, EGWG = excessive gestational weight gain, pre-pregnancy BMI at study start refers to the BMI measured before the pregnancy for which participants were recruited into the study

## Discussion

This study aimed to evaluate the impact of an mHealth intervention combining app-based self-monitoring and lifestyle coaching on EGWG among a cohort of 172 women who had previously experienced EGWG in their previous pregnancy. In contrast to our prior findings regarding self-monitoring via the INTER-ACT app and PPWR (Geusens et al., 2024), we did not find any associations between self-monitoring of weight, physical activity and mood, nor coaching attendance, and EGWG in the consecutive pregnancy. This indicates a limited effect of the intervention. Previously, we already found limited effects of the INTER-ACT intervention on PPWR, fat percentage and waist circumference when comparing women in the intervention arm and control arm (Van Uytsel et al., 2022). We now add that greater intervention adherence in terms of self-monitoring via the app and coaching session attendance did not reduce EGWG in a consecutive pregnancy in the INTER-ACT intervention. On the contrary, there is some evidence to suggest that both digital apps and face-to-face counselling can reduce gestational weight gain (Khademioore et al., 2025). This prompts consideration of whether additional face to face counselling or alternative componentsof the intervention were required, given that there is no clear consensus regarding the relative effectiveness of individual components. 

This highlights the need for continued research to identify which intervention components support weight management in pregnancy and the postpartum period, and which women are most likely to benefit from app-assisted lifestyle interventions.The literature suggests that some individuals have higher autonomous motivation and self-regulatory capacity regarding weight interventions compared with others (Paixão et al., 2020). Individuals with higher motivation and pre-existing healthy behaviours are often more likely to engage with and benefit from digital applications (O’connor et al., 2016), which is an important consideration when designing and targeting app-based interventions. In order to do this, future research should adopt and consistently use standardized weight (e.g., pre-pregnancy weight, gestational weight gain, postpartum weight retention) (Van Uytsel et al., 2023), motivational and mental wellbeing measurement across all phases of pregnancy. This approach would improve the quality, comparability, and clinical relevance of research into interventions for weight management during these critical periods.

We found that despite a positive initial field evaluation with high user acceptance of the intervention app (Bogaerts et al., 2020), user engagement decreased significantly between interpregnancy and the consecutive pregnancy. While most participants (78%) used the app to log their weight, steps and mental health during interpregnancy, only one in three did so during the consecutive pregnancy. This raises questions about the app design and participant characteristics that drive this reduced engagement. It seems that while the app is fairly attractive to postpartum women, it does not meet the needs of women who are pregnant again. Possibly, the combination of being pregnant while also having at least one young child at home, is reducing women’s engagement with an app (Bogaerts et al., 2020). The period of pregnancy is associated with increased contact with healthcare providers compared to postpartum and interpregnancy. A systematic review revealed that postpartum women valued behavior change strategies that were delivered through digital health interventions, however, lack of motivation and childcare priorities were a barrier to engaging with digital health (Lim et al., 2019). Thus, it is important to dedicate more research to understanding how we can optimize our interventions to fit into our participants’ changing lives. Women who discontinued their app use also attended fewer coaching sessions, indicating a general pattern of intervention discontinuation when comparing interpregnancy and consecutive pregnancy periods. Engagement declined markedly once participants became pregnant. One explanation may be that weight is routinely measured during prenatal visits, which may reduce the perceived need for additional self-weighing or app logging. Some studies have also reported that women may avoid frequent self-monitoring if gestational weight gain feels uncomfortable or exceeds expectations. It would be good to understand the types of apps women are using during pregnancy and postpartum to see whether the logging feature could be incorporated with a currently used app as a form to increase engagement. And consider engagement with apps over consecutive pregnancies. The second phase of the intervention began upto 15 weeks into pregnancy, by which point some women may have already gained more weight than recommended. This could contribute to decreased motivation and adherence to behavior change strategies (Leung et al., 2017). Additionally, pregnant women often feel that pregnancy is a time they are allowed to gain weight (Grenier et al., 2021).

In addition, weight remains a heavily stigmatized health issue, and preconception, pregnant and postpartum women are especially susceptible to this weight stigma (Hill & Rodriguez, 2020). Internalized weight stigma may initially encourage women to engage with the intervention, but may ultimately discourage them from continuing when they do not see the hoped-for results in their postpartum weight reduction. For these women, continued self-monitoring via the app may feel like being confronted with personal failure, which could contribute to the reduced engagement we observed in pregnancy. This aligns with our finding that participants who disengaged from the app also attended fewer coaching sessions. This would be in line with existing research on dietary lapses resulting in feelings of guilt and shame, increasing the risk of diet abandonment (Carels et al., 2004; Thøgersen-Ntoumani et al., 2021). More research is needed to better understand the role of weight stigma and the impact of weight management interventions in the perinatal phase on participants’ wellbeing and distress, and how this affects intervention adherence and effects.

Our findings that previous EGWG predicts future EGWG reinforce the importance of intervening early and supporting women prior to and between pregnancies (Waring et al., 2013). While our study focused on a digital intervention delivered postpartum and in early pregnancy, a broader body of literature highlights that sustained behaviour change may require support that begins earlier in the reproductive life course (Chong, 2022; Devine & Olson, 1991; Hector et al., 2012). Preconception or first-pregnancy interventions may be particularly valuable for women entering pregnancy with higher BMI or previous weight challenges (Mitchell et al., 2025). Future research could explore how app-based interventions might be integrated with preconception counselling, primary care contact, or community-level supports to better prevent EGWG and improve longer-term maternal health.

In our mHealth intervention, no measurable effect was observed. This highlights that behaviour change in pregnancy extends far beyond education alone; women’s choices are shaped by complex and inter-locking psychosocial, cultural, relational, economic and environmental factors (de Diego-Cordero et al., 2021; Michie et al., 2014). Eating and exercising are strongly influenced by culturally shared beliefs and family expectations -such as the idea that cravings signal what the baby “needs” -with reviews and cross-cultural studies showing that pregnancy cravings and food decisions are largely driven by cultural and psychosocial factors rather than biology (Sun et al., 2023) (de Diego-Cordero et al., 2021). These beliefs operate alongside practical constraints -household income, time available, and reliance on partner/family support -which can limit the feasibility of following guidance (Rebelle et al., 2022). In short, healthier eating and exercise in pregnancy requires multi-level approaches that attend to culture and family dynamics, partner involvement, and the everyday realities of money, time, safety and place, and reinforces that women need to have a community that supports her change in behavior between and during pregnancy.

An important consideration regarding our methodology is the potential limitation in detecting significant effects due to the small sample size. Based on the regression model in this secondary analysis (adjusted R² = 0.15, 11 predictors), the corresponding Cohen’s f² is approximately 0.18, indicating a medium effect size. With 172 participants, the study had adequate power to detect medium-to-large effects, but smaller effects of self-monitoring behaviors or coaching attendance on EGWG may not have been detected (Serdar et al., 2021). These null findings should therefore be interpreted with caution, and future studies with larger samples are warranted. Furthermore, maintaining participant engagement throughout the study proved challenging, which contributed to a smaller final sample size than originally intended, therefore we couldn’t conduct the stratified analyses comparing BMI groups that we originally planned (Harrell & Levy, 2022). Additionally, the mobile application used in the intervention presented several challenges. As a prototype, the app had gaps and technical issues, creating challenges for participants and extended delays in reporting and resolving these issues, which likely contributed to reduced engagement with the intervention. Nevertheless, early dropout is a well-known challenge in longitudinal studies. In an effort to minimize dropout, the INTER-ACT study provided participants with the flexibility to choose the location of their study visits (at home, in the hospital, or on location). Participants also received gifts, appointments at preferred times, and sessions supported by motivational techniques. We do not have (Headen et al., 2017) clear data on lost-to-follow up and withdrawal. However, baseline characteristics (e.g., age, BMI, anxiety, depression, PPWR) of participants included in this secondary analysis were similar to those of another subsample from the RCT (Geusens et al., 2024), suggesting that attrition was unlikely to have introduced systematic bias. Better data on this, along with qualitative research, would help to understand the reasons for dropout and the experiences of women. This will help develop more effective strategies to prevent participation attrition in future interventions. Furthermore, we did not have measures of pre-pregnancy weight, and have therefore used self reported, which is a common method (Bogaerts et al., 2012; Wuytack et al., 2023), as obtaining a measured pre-pregnancy weight is often difficult because many pregnancies are unplanned and not preceded by a clinic visit. While self-reported weight may have some error, this misreporting has not generally introduced significant bias into the associations between weight and birth outcomes (Headen et al., 2017), and therefore we have opted for this option.

In conclusion, there was a substantial decrease in the users of the app from interpregnancy to consecutive pregnancy, which potentially limited impact of the intervention. Future research is required with larger longitudinal interpregnancy samples improving the intervention engagement techniques.

## Data Availability

The data can be provided on request.

## Code Availability

Not availiable.
